# Understanding disease symptoms and impacts and producing qualitatively-derived severity stages for MPS IIIA: a mixed methods approach

**DOI:** 10.1186/s13023-022-02208-w

**Published:** 2022-02-22

**Authors:** Sally Lanar, Samantha Parker, Cara O’Neill, Alexia Marrel, Benoit Arnould, Bénédicte Héron, Nicole Muschol, Frits A. Wijburg, Anupam Chakrapani, Sophie Olivier, Karen Aiach

**Affiliations:** 1 Icon Plc, Lyon, France; 2 Lysogene, Neuilly-sur-Seine, France; 3 Cure Sanfilippo Foundation, Columbia, SC USA; 4 Pediatric Neurology Department, Center for Lysosomal Diseases, CHU Trousseau, APHP, Paris, France; 5 Department of Pediatrics, International Center for Lysosomal Disorders (ICLD), University Medical Center Hamburg-Eppendorf, Hamburg, Germany; 6 Amsterdam UMC, Pediatric Metabolic Diseases, Emma Children’s Hospital and Amsterdam Lysosome Center “Sphinx’’, University of Amsterdam, Amsterdam, The Netherlands; 7 Great Ormond Street Hospital, London, UK

**Keywords:** MPS IIIA, Qualitative research, Thematic analysis, Natural history study

## Abstract

**Background:**

MPS IIIA is a rare, degenerative pediatric genetic disease characterized by symptoms impacting cognition, mobility and behavior; the mean age of death is around 15 years of age. Currently, there are no approved therapies for MPS IIIA.

**Methods:**

A two-year, multi-center, prospective, descriptive cohort study was conducted to document the natural history course of MPS IIIA. In the context of this study, semi-structured interviews were performed with parents of children at study entry and one year later. Interview transcripts were analyzed using thematic analysis methods to identity concepts of interest to children and parents, identify what factors impacted parents’ burden the most, and develop qualitatively-derived disease severity stages. Children were sorted into these stages according to the symptoms their parents described at the entry interview. This sorting was compared quantitatively to the sorting of children at baseline according to the child’s calendar age and their BSID development quotient (DQ).

**Results:**

22 parents in France, Germany, the Netherlands and the UK were interviewed. Children ranged in age from 28 to 105 months (mean 61.4 months). The conceptual models for children’s symptoms and impacts and parents’ impacts provided a detailed and comprehensive picture of what it is like for children of various ages and their parents to live with MPS IIIA. Four factors were identified as mediating the burden perceived by parents: state support, family support, time since diagnosis, and parent coping strategy. Four disease stages were developed, accounting for both the presence and the severity of MPS IIIA symptoms. The comparison of children’s sorting into these stages with the BSID DQ and the child’s calendar age showed strong statistical associations.

**Conclusions:**

The findings of this qualitative research embedded in a natural history study add to the current understanding of MPS IIIA as a complex disease that impacts every aspect of the lives of children and their families. This study demonstrates the unique potential of mixed methods research in rare diseases to address some of the current limitations of more traditional quantitative approaches by providing an individualized, detailed understanding of the patient experience.

## Background

MPS IIIA is a rare pediatric genetic disease with an estimated prevalence of between 1:50,000 and 1:250,000 depending on the population studied [1]. MPS IIIA typically manifests with severe neurodegeneration combined with less prominent but present somatic symptoms. The majority of children present the rapidly progressing or severe classical form of MPS IIIA, typically first detected by clinicians when the child is between one and three years of age and shows slowed cognitive development, followed by severe behavioral problems, loss of speech, and progressive intellectual decline. Later in the teenage years, children experience onset of severe dementia and decline of all motor functions culminating in the loss of locomotion, dysphagia, and spaticity/clonus [2–4].The mean age of death is about 15 years of age [5]. The most commonly reported cause of death is pneumonia [6]. There is currently no disease modifying treatment for MPS IIIA [7].

A two-year, multi-center, prospective, descriptive cohort study was conducted to document the natural history course of MPS IIIA and to obtain standardized assessments of not only neurocognitive development, but also behavioral capabilities, sleep–wake habits, pain, eating behavior, and the effect of MPS IIIA on the health-related quality of life (HRQOL) of children and their families. Due to the complexity of the disease and the variability of its expression, quantitative HRQOL assessments are not anticipated to be able to capture all meaningful aspects of the children’s and families’ experiences. As such, semi-structured interviews were also conducted with parents of children at baseline and at Month 12.

The objectives of the present paper are three-fold. First, we describe the symptoms and impacts of MPS IIIA on children and their parents, including the factors that influence parents’ burden. Second, we explain the qualitative development of a four-stage description of disease progression and the sorting of children in the natural history study into these stages based on collected data. Third, we examine how the four-stage classification of children’s disease progression compares with classifications based on the Bayley Scales of Infant Development and the child’s calendar age.

## Methods

### Study design

The semi-structured interviews were part of a multi-center, prospective, descriptive cohort study on the natural course of MPS IIIA. Standardized clinical and observer-reported neurocognitive, developmental, and behavioral measures were captured at baseline and every 6 months for up to 24 months. These measures included the Bayley Scales of Infant Development, Third Edition (BSID-III).

All parents of children included in the study were invited to participate in interviews at baseline and at Month 12; participation was voluntary. Parents needed to have completed a baseline interview to be eligible for the Month 12 interview. Interviews were conducted in France, the Netherlands, Germany, and the UK and took place within three months following the baseline and Month 12 site visits.

Up to 25 MPS IIIA children were anticipated to be enrolled. To be eligible, children had to be nine years of age or younger, have a documented MPS IIIA diagnosis, be medically stable, and have never received an investigational product for MPS IIIA.

### Interview conduct and transcription/translation

Interviewers were trained on the background of MPS IIIA and the natural history study, the use of the interview guide, and interviewing methodology.

Face-to-face parent interviews took place at a location of the parent's choice. All interviews were done by local interviewers in the parents’ native language; all Month 12 interviews except for two were conducted by the same interviewer as the baseline interview. Both baseline and Month 12 interviews lasted one hour to one hour and half.

During the interviews, parents were asked to identify which symptoms were the most challenging to manage; to discuss how caring for their child and managing their symptoms affects the family; and to describe how their child's symptoms have changed since diagnosis for the baseline interview or since the baseline interview for the Month 12 interview.

Interviews were audio-recorded with parents' consent and were transcribed word-for-word in the source language and de-identified. At baseline, German and Dutch transcripts were translated into English for analysis; English and French interviews were analyzed in the source language. At Month 12, no translations of transcripts were done; all interviews were analyzed in the source language.

### Qualitative analysis

#### Conceptual models describing symptoms and impacts of children and parents

The qualitative analysis of the baseline interviews that led to the development of the conceptual models was an exploratory, iterative process. Concepts were organized into themes representing higher-order categories of analysis. The themes were then sorted into one of two conceptual models: either the model for children’s symptoms and impacts or the model for parent impacts.

Analyses for conceptual models were performed using a qualitative software package, ATLAS.ti Version 8. ATLAS.ti is designed to facilitate the storage, coding, analysis, and retrieval of qualitative data [8].

#### Qualitatively-derived severity stages

The qualitatively-derived severity stages were developed exclusively based on the symptoms described by parents of children with MPS IIIA during the qualitative interviews. No other data on children (such as age, gender, country, clinical or questionnaire data from the natural history study) was used to inform their development.

The development proceeded by inductive reasoning using the conceptual models as a starting point. The most common symptoms found in the sample were considered as the core of the disease and were grouped in Stage 1. Stages 2 and 3 were conceived as a progression of symptoms already present in Stage 1 as well as the accumulation of new symptoms. Stage 4 was conceptualized as reflecting the most severe, and often most uncommon, symptoms found in the sample. As a whole, the stages were developed to reflect MPS IIIA symptom progression (the worsening of a current symptom, such as loss of language), symptom accumulation (the addition of new symptoms alongside prior ones, such as motor function issues in addition to trouble self-feeding), and the emergence of critically severe symptoms (such as trouble with nutrition).

Following the definition of these four stages, each child was sorted based on an in-depth review of the child’s baseline transcript and an examination of the codes that were used in that transcript. Children were sorted into stages in a holistic, qualitative fashion. In other words, a child did not need to systematically have *all* of the symptoms in a stage to be sorted into that stage description.

### Mixed methods: triangulating qualitatively-derived severity stages with BSID-III Development Quotient and child’s calendar age

We compared the sorting of children according to the qualitatively-derived severity stages, the child’s calendar age at baseline (6 years and older vs. less than 6 years old), and the child’s baseline BSID-III development quotient (DQ). DQ cognition was defined as mild/normal if 75–100%; moderate if 50–75%, and severe if < 50%. DQ is calculated by dividing the development age into which test scores place the child by the child's chronological age and multiplying by 100 [9]. The statistical associations were tested with Mantel-Haenzel Chi^2^ exact test.

## Results

### Interviewed population

The parents of 22 children were interviewed at baseline in Germany, France, the Netherlands, and the UK. Of these 22 parents, 16 were also interviewed at Month 12. The baseline interviews were completed between July 2016 and June 2017. The Month 12 interviews were completed between September 2017 and May 2018. Sociodemographic data on the children and their parents are presented in Table 1.

**Table 1:** Children’s and parents’ sociodemographic data

Category	At baseline interview (N = 22)
Child’s age at baseline site visit (months)	
Min–max	28–105
Mean	61.4
Country (n)	
France	8
UK	3
Germany	6
Netherlands	5
Child gender (n)	
Male	16
Female	6
Parent interviewed (n)	
Mother only	9
Mother and father	12
Mother and friend	1
Child’s position in family (n)* *Children’s half-siblings as considered in the determination of a child’s position in family	
Only child	5
Oldest in family	6
Youngest in family	7
Middle child	4
Parents’ civil status (n)	
Living with a partner	21
Single	1
Parents’ employment status (n) [N = 33]* *Number of parents present at the interview excluding “friend” N = 33	
Full time	11
Part time due to child	5
Part time not due to child	6
Unemployed due to child	7
Sick leave	3
Maternity leave	1

### Conceptual models for children’s symptoms and impacts and parents’ impacts

**Fig. 1:**
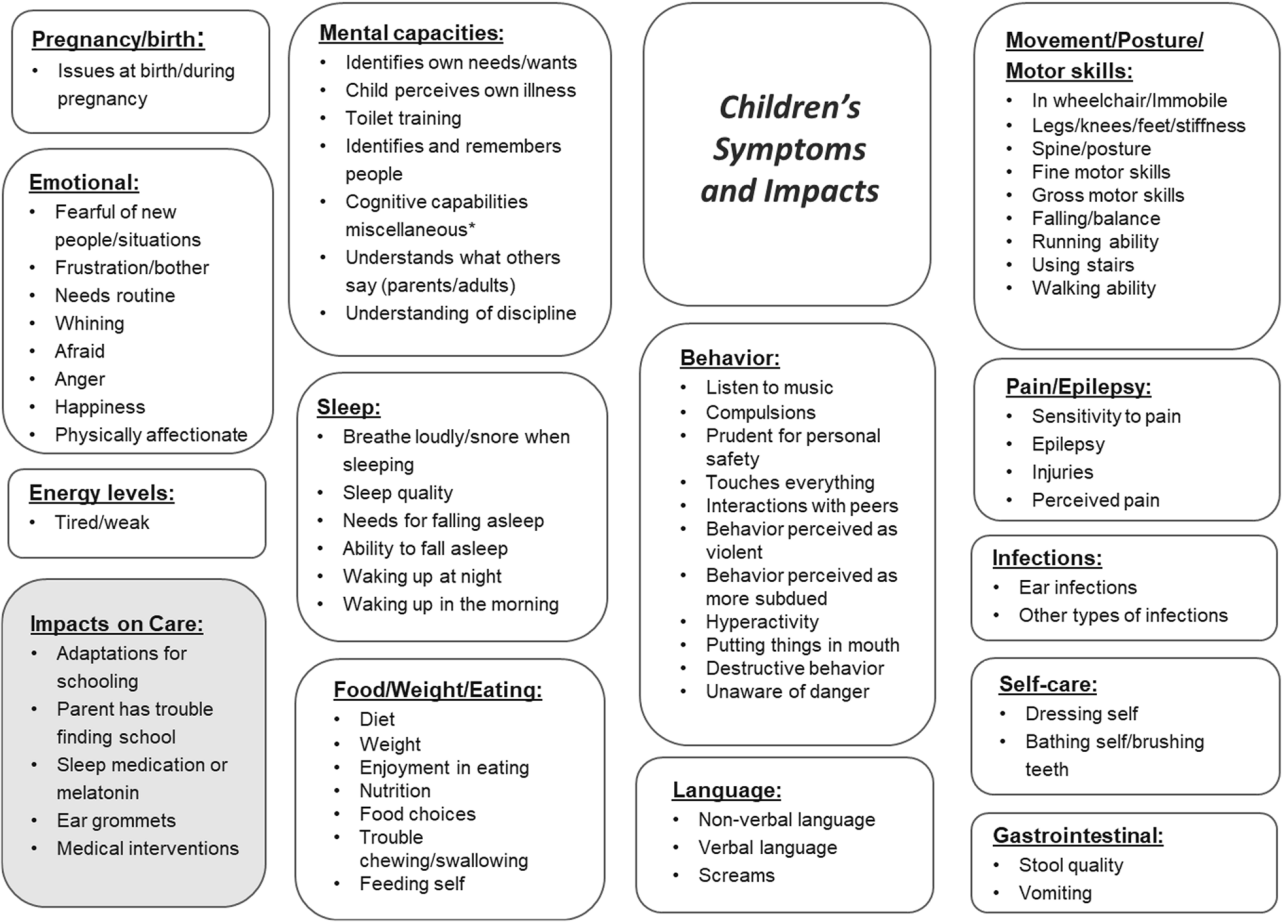
Baseline conceptual model of children's symptoms and impacts

The conceptual model for children’s symptoms and impacts (Fig. 1) provided a detailed and comprehensive picture of what it is like for children of various ages to live with MPS IIIA. In general, the model showed that MPS IIIA is an illness which impacts multiple aspects of a child’s health and HRQOL. In terms of symptoms, the model covered symptoms related to behavior, mental capacities, sleep, movement/motor skills/posture, emotions, birth conditions, development, self-care, pain, epilepsy, language skills, eating habits, weight, energy, gastrointestinal health, infections, and disease severity as evaluated by the parents. In terms of impacts, the model covered schooling options and medical interventions. Illustrative quotes for selected symptoms and impacts are presented in Table 2.

**Table 2:** Illustrative quotes for conceptual model of child's symptoms and impacts

Theme	Concept	Quote
Behavior	Hyperactivity	“He’s always like Jack Flash, fast as lightning. […] If you say slow down, he gets really excited. He’s always full of energy” (Patient 101)
	Unaware of danger	“When he’s excited, it’s too late. Then he also runs ahead and scurries about and talks to everyone he meets on the street. It’s […] definitely takes an effort to keep an eye on him then. So that he won’t simply run away somewhere” (Patient 305)
Sleep	Wakes up at night	“Last night I think he woke up about half past 11, then I got him back down, gave him a drink, then he woke up at like 2. The night before he was literally awake from half past two till about five o’clock in the morning, and then he went to sleep again, and then you’re having to wake him up because he’s tired” (Patient 103)
	Trouble to fall asleep	“We left her in her bedroom so that she would go to sleep by herself like any child, but […] she would not go to bed right away. She would go to bed late. So, the next morning, she was tired, obviously” (Patient 405)
Speech/ language	Verbal language	“He can’t talk, he’s never really been able to talk. […] He can sing, but he, sort of, sings the tune rather than the actual words.[…] If you asked him a question or something he wouldn’t really know what you mean. Well, if you say to him, do you want a biscuit, and if you show him the biscuit, he’ll understand what that is, he might point to something but that’s about it really” (Patient 102)
	Non-verbal language	“Speaking is still very hard for him even today. […] But he shows more with his hands what he wants. Yeah, with gestures he expresses what he wants. But he can't speak. Not the way he should” (Patient 303)
Pain	In pain	“She has often been hospitalized when she was in severe pain. So, she was given Valium […] When things aren’t going well, I call them [the hospital] and I say, ‘Take her for a three-day in-patient stay, under surveillance,’ and I stay with her, alone, for several days, while the dad stays here [with the other children]” (Patient 407)
	Sensitivity to pain	“It’s true that, it’s annoying, these children [with MPS IIIA], they don’t complain…you need to guess! You need to guess where they are in pain! […] It’s frustrating” (Patient 402)
Motor skills	Walking	“He does have trouble walking. […] He doesn’t walk, he has two speeds; one’s running and one’s standing still […]. He’s got problems with his legs so he is a little bit unstable. […] So if we go out we can’t let him—we just can’t let him walk on his own, because for one he’d just run off and two, he hasn’t—he’s just not aware of what’s happening around him” (Patient 103)
	Running	“He walked quickly. It was never running. But like the physiotherapist said, running was more for safety. The faster you move, the sturdier the movement. So, for him it was harder to walk. That's why he always ran. Of course, he always stumbled over something. Since you have to look where you're going” (Patient 302)

**Fig. 2:**
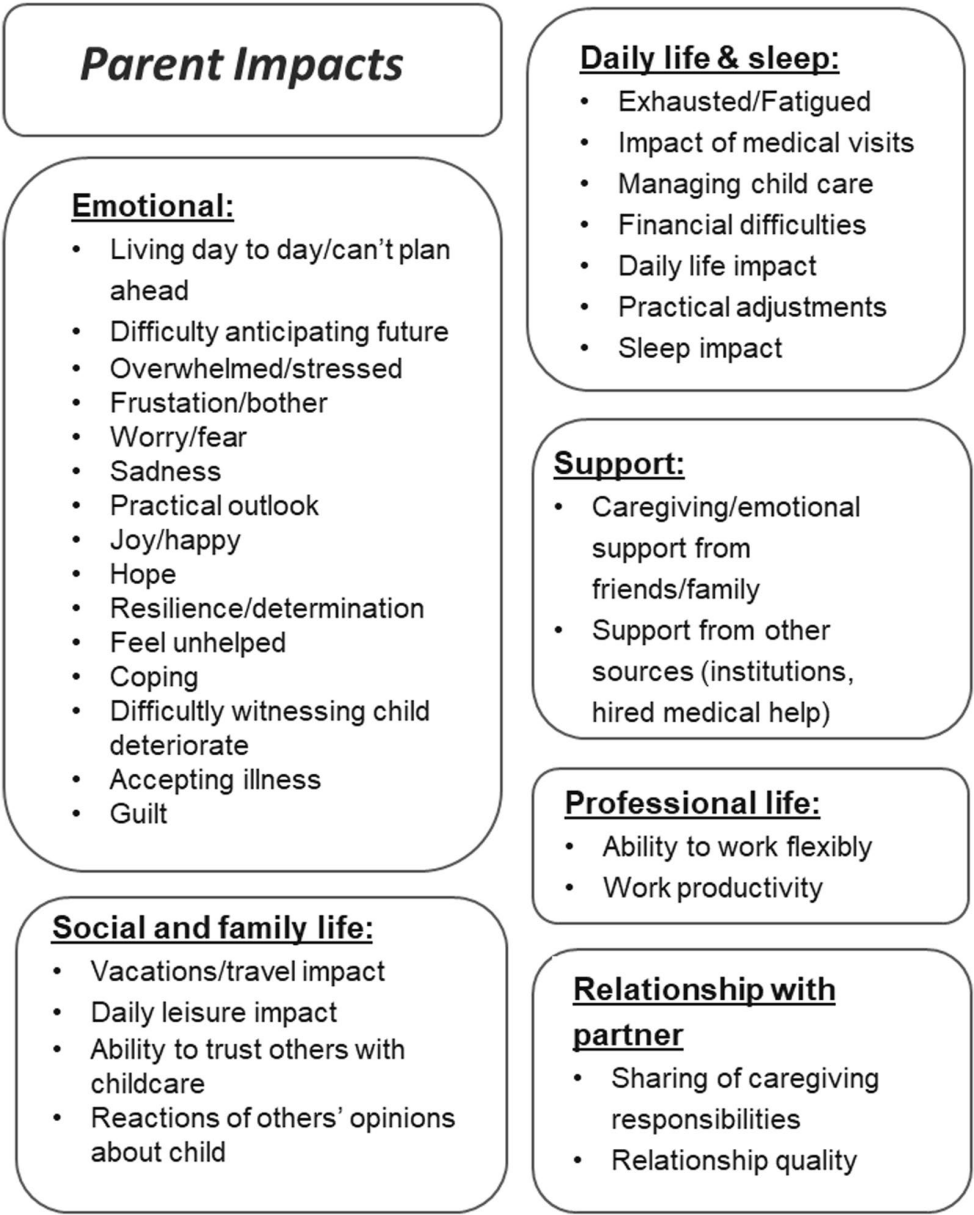
Baseline conceptual model of parent impacts

The conceptual model for the impact on parents (Fig. 2) showed that to be a parent of a child with MPS IIIA is to have your life impacted in every aspect. The emotions parents feel are diverse and powerful, ranging from depression and anxiety to joy and determination. Parents’ activities of daily living are radically transformed as are their sleeping habits. The relationships between the parents and between the parents and their friends and relatives change. Parents’ professional life, such as finding and keeping employment and professional satisfaction and success, are similarly affected. Illustrative quotes for selected impacts are presented in Table 3.

**Table 3:** Illustrative quotes for conceptual model of parents' impacts

Theme	Concept	Quotes
Emotional impact	Difficulty anticipating future	“[Child name]’s young, there’s a lot of positive things happening in the world, we focus on that. We’ll deal with what comes when it comes, I don’t want to think about it, because I hope it won’t. […] I think [my partner] struggles with more of the MPS side, what’s going to happen” (Parent of patient 101)
	Frustration/bother	“There wasn't anything I could do, because the screaming was really [bad] … And our neighbors could hear the screaming. […] There was a bar with a terrace in front opposite our house. […] those people knew exactly what was going on. […] That is very frustrating” (Parent of patient 201)
		“Sometimes when it's too much, I say: ‘Yes, you've got it…’ because it's too much even for me. Because he cries, he screams, he hits others, he hits himself, breaks things. […] There are moments when I give up” (Parent of patient 303)
	Overwhelmed/stressed	“So, what bothers me is that every minute of my day is filled up. I also like to sit down and have a coffee. And I don't have time for this. Because I'm always racing from appointment to appointment and errand to errand. Once a week I have physiotherapy with [child’s name], then I have speech therapy with [child’s name]. Then I go with [sibling] to gymnastics, [sibling] can't be short changed either. She [the child’s sibling] is important too after all. And all of this always right after work” (Parent of patient 301)
	Worry/fear	“I also talk a lot with other MPS parents. […] we regularly go to MPS meetings and right before that I worry about it a lot. And it's really very important to exchange ideas with the parents and gather experiences. So, that's the time when I worry about it a lot. But during my daily routine I don't have any time at all to worry about what might happen in 2 years” (Parent of patient 301)
		“What scares me the most is that he will be in a wheelchair because the majority of them [children with MPS IIIA] will find themselves in a wheelchair. I tell myself, today, he has lost some of his language skills. Well, we communicate differently, it’s not a problem. It’s more, […] the fact that he would be in a wheelchair, that, that would destroy me because I couldn’t handle it” (Parent of patient 402)
Support	Caregiving support	“You don't let just anyone watch him because you don't want to expect that much of just anyone. […] So, there's someone in my circle of friends that you can count on. That is, if there are things they can help with, they do it. In terms of friends, we can't complain. Then there are some others who say: ‘We can't handle the situation’” (Parent of patient 302)
		“My mother and father are also there. And his [my partner’s] parents are also there, but it seems as if they don’t really get the situation completely. They know that [child’s name] is ill and ultimately, we are the ones who spend the most time with him. It’s simply too tough to spend a whole day with [child’s name]. Our parents are also at the age that this isn’t easy for them. They are definitely there to provide emotional support. I provide the care for [child’s name] and am also not happy to delegate it” (Parent of patient 202)
Social and family life	Daily leisure	“Some things are a bit more tricky to take him to because he might get upset and start crying, he doesn’t want to sit down and sit still, if we go to a restaurant for dinner, go to a pub or something like that, he doesn’t want to sit at the table and sit still, he’ll keep getting down, running around and that sort of thing, so we’re a bit more conscious now, but we still try and do most things that we did before” (Parent of patient 102)
Work impacts	Work	“It’s [child having MPS IIIA] affected her [my partner’s] work, she has to have time off work when he’s not well and hospital appointments and […] if nursery phone up and say, ‘Look, he’s not well,’ or he’s got a rash or whatever […], we’re a bit—[parent mimics discussion between him and his spouse] ‘Like I can go and pick him up, or can you pick him [up]?’—‘I can’t today I’m in a meeting, I can’t get out of it’” (Parent of patient 103)
Daily life & sleep	Sleep	“We had decided it [to put the child in an institution] then as she awoke 10 times per night. I must get out of bed 10 times per day. She awoke our son [her brother], she awoke our entire house. At any given moment, you sit here and you just cannot anymore” (Parent of patient 205)
		“Especially the initial period was very much a period of… Not even the sleeping as such, but… It just eats away at you. You are not yourself. I am still noticing that I'm more irritable. I go to bed and sleep well. I've slept even since getting the diagnosis. Even when I kind of felt: how can this be? I still didn't mull things over in bed. I went to bed, fell asleep but would then wake up again. That was not fun. But well… It is what it is” (Parent of patient 201)

### Factors influencing the disease burden of parents

The global impact on parents caring for a child with MPSIIIA was mostly mediated by the following four factors:

1. State-funded support from local or national government in the form of specialized schools, transportation to and from medical professionals/schools, and/or financial aid for medical expenses. For example, if parents struggled to find a school that would accept a child with a higher level of need or if the school was far away from a parents’ home and did not provide transport of the child, this could increase parents’ stress and time burden.
2. Child-caretaking and emotional support from the parents’ families. Child-caretaking was more helpful than emotional support for parents. Parents were reluctant to ask for help from outside of the family; few felt comfortable asking neighbors or babysitters to watch their children. Parents were also reluctant to place their child in a specialized full-time care institution because they considered it their responsibility to provide for their child’s care.
3. Time since MPS IIIA diagnosis: Parents who were interviewed at baseline in the months following the diagnosis were often processing feelings of shock, depression, anxiety, and anger during this time. Parents whose baseline interview took place after their child had been diagnosed for several years had accepted their child’s illness and were more focused on maintaining function and alleviating perceived suffering. Their concerns were more practical, for example, decisions on which medical equipment to purchase or how to renovate their home in light of their child’s reduced mobility.
4. Coping strategies of parents: The coping strategies parents described adopting in the face of their child’s illness varied across the sample. One coping strategy parents put in place was to adjust their perspective on the illness: instead of conceiving of the illness as a tragic life event, parents chose to view it an opportunity to re-evaluate their life choices. Another coping strategy consisted of choosing to balance child-care time with other activities the parents did individually or as a couple, such as professional responsibilities at the office or hobbies like sports.

### Qualitatively-derived severity stages

**Fig. 3:**
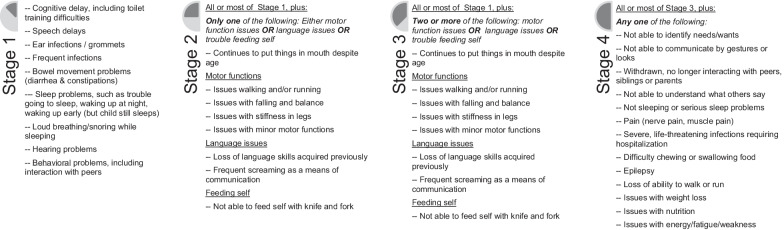
Qualitatively-derived severity stages for MPS IIIA

The qualitatively-derived severity stages are shown in Fig. 3. Three children were categorized in Stage 1, seven children in Stage 2, six children in Stage 3, and six children in Stage 4.

### Comparison of qualitatively-derived severity stages with BSID-III Development Quotient and child’s calendar age

The comparison between (1) the qualitatively-derived severity stages, (2) the BSID-III DQ, and (3) the child’s calendar age (below 6 years old vs. 6 years old and above) showed a very good statistical association between the qualitatively-derived severity stages, the BSID DQ, and the child’s calendar age (Table 4).

**Table 4:** Cross-table between qualitatively-derived severity stages, baseline BSID-III DQ, and baseline child’s calendar age (N = 22)

Variables	Qualitatively-derived severity stages				
	Stage 1 (N = 3)	Stage 2 (N = 7)	Stage 3 (N = 6)	Stage 4 (N = 6)	*P* value^[a]^
BSID-III DQ					0.0126
Moderate (DQ 50–75)	2	4	1	0	
Severe (DQ < 50)	1	3	5	6	
Age at baseline (years)					0.0008
Below 6 years old	3	7	4	1	
6 years old and above	0	0	2	5	

^[a]^*p* value from Mantel–Haenszel Chi^2^ exact test

Children with moderate cognitive loss as per BSID-III DQ at baseline were mostly categorized in Stage 1 and 2 (n = 6 out of 7 children). Conversely, children with severe cognitive loss at baseline were mostly categorized in Stage 3 and 4 (n = 11 out of 15 children).

Stage 1 and 2 only contained children who were below 6 years old. Four children in this age group were in Stage 3 and only one in Stage 4. Children 6 years and older were only in Stage 3 and 4, with most children in this age group in Stage 4.

The comparison between BSID-III DQ and the child’s age showed that the two measures were somewhat associated. All children above 6 years old were classified as experiencing severe cognitive loss (Table 5).

**Table 5:** Cross-table between BSID-III DQ and the child’s calendar age at baseline (N = 22)

Variables	BSID-III DQ by class		*P* value^[a]^
	Moderate cognitive impairment (N = 7)	Severe cognitive impairment (N = 15)	
Age at baseline (years) by class			0.0513
Below 6 years old	7	8	
6 years old and above	0	7	

^[a]^*p* value from Mantel–Haenszel Chi^2^ exact test

## Discussion

In contrast to other qualitative research conducted in MPS III [10–12], our study consisted of interviews embedded in the context of a natural history study focused exclusively on MPS IIIA European children and their parents. These interviews were longitudinal, lasted approximately one hour and were often conducted with both parents of the child present. The results of this unique methodology included a detailed picture of MPS IIIA in the form of two conceptual models, one for the child’s symptoms and impacts and one for parent impacts, an understanding of the factors that influence parents’ disease burden, and the qualitatively-derived severity stages.

Parents in our study described a multi-faceted disease in which non-cognitive symptoms (e.g., infections, sleep, pain, mobility) could be as impactful on parents and children as cognitive symptoms (e.g., behavior, language). These findings reinforce recent recommendations and research on outcomes measurement in MPS IIIA which focus on the need to consider other aspects of the disease in addition to cognitive symptoms [10, 13–15].

The themes and concepts in our models complement those that have been published in the recent literature on MPS III [10–12]. Indeed, the most important symptoms parents identify to treat in MPS IIIA (speech/loss of language, walking/running, sleep, eating, pain, and hyperactivity) are part of the conceptual models and the qualitatively-derived severity stages (Cara O’Neill, personal communication, [11, 12]). Parents also discuss these symptoms in the FDA listening sessions on MPS III [14, 15].

Our study provides insights into the factors that influence parents’ experiences with MPS IIIA and highlights that multiple factors beyond disease severity are important modifiers to the global disease impact parents feel. Receiving state support (logistical and financial) and family support (emotional and care-taking help) lessens the demands placed on parents. The older the child is, the more likely parents are to have emotionally accepted the diagnosis and be focused on practical day-to-day disease management. The coping strategies parents employ also shape how they experience their child’s illness.

A unique result of this research was the qualitatively-derived severity stages. In line with recent recommendations and research on MPS IIIA, these stages evaluate the presence and progression of both cognitive and non-cognitive symptoms of MPS IIIA [10, 13–15]. An encouraging finding of the severity stages is the existence of a clear statistical association between the ranking of children according to these stages, the child’s calendar age, and the BSID-III DQ. On the one hand, we have a qualitative assessment based on semi-structured interviews and thematic, inductive analyses, and, on the other hand, a quantitative assessment based on a standardized cognitive assessment tool. This finding opens the door for improvement in patient-centricity research in rare and ultra-rare diseases where the development and validation of disease-specific patient-reported outcome scales is limited [16–19].

Previous research has presented MPS IIIA in three stages of age-dependent disease development [20]. These three stages do not capture the heterogeneity of the disease where children progress at different speeds and do not all have the same problems at the same time [20, 21]. The four qualitatively-derived stages may address some of the limitations of this three-stage model. Indeed, the lived experience of parents demonstrates that separating the middle stage from the original three-stages model into two distinct stages is helpful, particularly since children have differences in which body systems (e.g., motor function, language or self-feeding) are affected first (Cara O’Neill, personal communication).

The conceptual model of children’s symptoms and impacts and the qualitatively-derived stages both include pain as a symptom of MPS IIIA. However, it is difficult to determine how accurately the models and the stages reflect the child’s experience of pain. Recent literature on pain in MPS III and in central nervous system diseases in children suggests that parents and medical professionals alike often misunderstand pain and that pain could be the cause of various behaviors in MPS IIIA [22–24]. As such, it is possible that the parent-reports of pain on which our analysis relies misrepresent this symptom from the child’s viewpoint.

Although regulatory bodies increasingly recommend qualitative research [25], it, like all types of research, comes with its own limitations, in particular the subjectivity of the interviewers and the researchers who perform the qualitative analysis. In this study, we strove to balance these limitations in two ways. First, by employing trained, experienced interviewers who conducted the interviews following the same semi-structured guide in each country. Second, by triangulating the ranking of the children according to the qualitatively-derived severity stages with clinical measures (the BSID III DQ and the child’s calendar age). The strong association between these clinical measures and the qualitatively-derived severity stages reinforces the robustness of the qualitative research reported here.

## Conclusion

The findings of this qualitative research embedded in a natural history study complement the current understanding of MPS IIIA as a multifaceted disease that impacts every aspect of the lives of children and their families.

It provides new insights into the factors that influence the degree of parents’ burden: state support, family support, time since diagnosis, and parent coping strategy. The more state support and family support parents receive, the better they are able to handle the emotional and logistical demands of MPS IIIA. The more time passes since diagnosis, the better parents adjust to the emotional burden of the illness.

Beyond describing the symptoms and impacts of MPS IIIA, this qualitative research proposes a new way of conceptualizing of disease progression in the form of four severity stages combining cognition, behavior, language, motor skills, pain, sleep, eating habits, and other aspects of MPS IIIA. These stages enable a qualitative classification of children in the natural history study and reflect clinical differences between children found in the BSID-III DQ and the child’s calendar age.

From a broader viewpoint, this study demonstrates the unique potential of mixed methods research in rare diseases. Mixed methods address some of the current limitations of more traditional quantitative approaches by providing an individualized, detailed understanding of the patient experience.

## Data Availability

Any requests concerning the availability of data and materials should be addressed to Lysogene.

## Abbreviations

MPS IIIA: Mucopolysaccharidosis type Ill A
BSID-III: Bayley Scales of Infant Development, Third Edition
DQ: Development quotient
HRQOL: Health-related quality of life
